# 

**Published:** 1895-12

**Authors:** 


					﻿Vol. XV.
DECEMBER, 1895.
NO. 12*
THE
pOM(EOPATHlC PHYSICIAN-
A Monthly Journal of Homoeopathic Materia Medica
and Clinical Medicine.
" He is a freeman whom truth makes free, and all are elates beside."
."Seek the Iruth: come whence it man, cost what it will."
EDITED AND PVBLIBHED BY
WALTER M. JAMES, M. D.
PHILADELPHIA:
JSTo. 1231 LOCUST STREET.
- /-	LONDON :
ALFRED HEATH & CO., 8olb Agent# fob Great Britain,
114 Ebury Street, Eaton Square.
Yearly Subscription, $2.50, invariably in advance. Sinyle numbers, 25 cis.
Entered at the Philadelphia Poet-Offioe ae Saeond-ClaM Mail Matter.
				

